# Post abortion family planning counseling as a tool to increase contraception use

**DOI:** 10.1186/1471-2458-9-20

**Published:** 2009-01-15

**Authors:** Ali Ceylan, Meliksah Ertem, Gunay Saka, Nurten Akdeniz

**Affiliations:** 1 Department of Public Health, Medical Faculty of Dicle University, Diyarbakir, Turkey; 2 Department of Obstetrics and Gynecology, Medical Faculty of Dicle University, Diyarbakir, Turkey

## Abstract

**Background:**

To describe the impact of the post-abortion family planning counseling in bringing about the contraceptive usage in women who had induced abortion in a family planning clinic.

**Method:**

The Diyarbakir Office of Turkish Family Planning Association (DTFPA) is a nonprofit and nongovernmental organization which runs a family planning clinic to serve the lower socio-economic populations, in Diyarbakir-Turkey. Post abortion counseling is introduced by using proper communication skills and with using appropriate methods to women. In this study we introduced contraceptive usage of women who had induced abortion one year ago and followed by DTFPA's clinic.

**Results:**

55.3% of our clients were not using contraceptive methods before abortion. At the end of the one year, 75.9% of our followed-up clients revealed that they were using one of the modern contraceptive methods. There was no woman with IUD before induced abortion. At the end of one year 124 (52.3%) women had IUD. "A modern method was introduced immediately after abortion" was the most important factor increasing modern method usage.

**Conclusion:**

Our results advocate that post-abortion counseling may be an effective tool to increase the usage of contraceptives. Improved and more qualified post-abortion family planning counseling should be an integral part of abortion services.

## Background

The World Health Organization (WHO) estimates that, worldwide, almost 20 million unsafe abortions take place each year, with 95% of these performed in developing countries. About 80,000 maternal deaths per year are thought to be due to abortion complications, accounting for about 13% of all maternal deaths in the world, one in eight pregnancy-related deaths [1]. An estimated 123 million couples, mainly in developing countries, do not use contraceptives, despite wanting to space or limit their childbearing [2]. Post-abortion counseling incorporated into post abortion care has been regarded as an appropriate venue or vehicle to decrease unwanted pregnancies and induced abortions [3].

Abortion services have been readily available in Turkey since 1983, when it was first legalized. According to the family planning law (1983), induced abortions can be performed to pregnancies not exceeding 10 weeks. In Turkey, 12.4% of the women have had at least one induced abortion [4]. Most of the public hospitals and maternal health clinics perform abortion; however, these services are mainly provided without sufficient family planning counseling. Turkish National Health Survey (TNHS) reported that 42.5% of the women have used a modern contraceptive method while 28.5% of them have used traditional methods, and 40% of the women had unmet family planning need [5].

Our study was conducted in Diyarbakir, which is considered to be an underdeveloped province in Turkey. The availability of health facilities and the utilization of family planning are low in the province [6]. In this city, only 28.1% of the women have used one of the modern contraceptive methods and most of the women had no chance to reach the abortion services [7,8]. The Diyarbakir Office of Turkish Family Planning Association is one of a organization which introduce induced abortion services in slums of Diyarbakir.

The Diyarbakir Office of Turkish Family Planning Association (DTFPA) is a nonprofit and nongovernmental organization which runs a family planning clinic to serve the lower socio-economic populations, in Diyarbakir and all health services are free of charge. In our clinic, the nurses and physicians work voluntarily and were trained in counseling methods by professional trainers from the Dicle University. Post abortion family planning counseling was provided to all clients attending our clinic for medical abortion.

In this study, we aimed to evaluate the effect of post abortion family planning counseling on contraceptive usage. Beside this we tried to answer the question of why the women did not use contraceptive methods although they had life training induced abortion.

## Methods

### Subjects

The records of 322 women who underwent induced abortion and who had been provided post-abortion counseling between May 2003 and April 2004 were the main source of this study. One year after the abortion date women were invited to clinic by telephone call or periodic home visits. But only 237 women could be reached, followed and interviewed. Sixty two women could not be reached, and 23 women reluctant to participate in the study. Interviews were carried out with emphasis on the age of women, marital status, education level, attitudes and practices towards contraception.

The study carried out in compliance with the Helsinki Declaration. Only women who gave informed verbal consent were included in the study. The Investigators were aware of the ethical, legal and regulatory requirements for research on human subjects in Turkey as well as applicable international requirements. We obeyed the Turkish legal rules for performing induced abortion.

### Study Setting

The study conducted in Diyarbakir which is a city in the least developed region of Turkey. Illiteracy rate in women was 44% in Diyarbakir [9]. DTFPA's clinic was settled in suburban area of Diyarbakir so the situation women were worse.

### Intervention and training of health staff

In accordance with the goal of our Association, appropriate post abortion family planning counseling has been provided to all of our clients. Post abortion counseling included proper communication skills of our providers and application of principles in adults learning. The principles that we tried to apply were that adults learn easier if the subject is relevant to their interest and previous experiences, if their feelings and attitudes are identified, and if they are actively involved in the learning process. Various kinds of teaching techniques were used [10]. Three physicians and seven nurses were trained on adult training principles by a trainer from Dicle University. Physicians and nurses were also trained on good interpersonal communication skills, including the ability on effective questioning, active listening, summarizing, paraphrasing clients' comments or problems, and adopting a non-judgmental, helpful manner. The training session of physicians and nurses took two weeks (40 hours), in which, half of the lectures were based on practice.

### Applications

Counseling was conducted in a private room, with sufficient time (20 to 45 minutes) and confidentiality assured. We tried to help our clients to understand their reproductive legal rights, to learn to reach their self determined goals through well-informed choices. Information about modern contraceptive methods was given. Condoms, combined oral contraceptive pills and intra uterine device (IUD) were introduced by physicians and nurses of our clinic. We followed up our clients by telephone calls or by home visits to take information on their complains about the chosen contraceptive method. After the abortion, each woman visited in her home with 6 months intervals, women that we couldn't find her at home interviewed by telephone call. Trainers from Dicle University also supervised physicians and nurses of DTFPA on the manner of proper counseling rules mentioned above. If a woman decided to tubal ligation or other methods that were not available in our clinic, we referred them to public maternal health clinics or to the University Hospital.

### Statistical Analysis

The information obtained was recorded and analyzed by using EpiInfo 2000 (CDC, USA) computer program. We dicotomised modern contraceptive usage (yes or not). To show the effect of proper counseling on contraception usage, adjusted odds ratios were calculated for age, education level, parity, and desire for future fertility. The odds ratio and 95% CI for not using contraception were calculated by using logistic regression analyses.

## Results

### Demographic and fertility features

Age groups were similar in three different groups (p = 0.164). Illiteracy rate was 51.9% (123 women) (Table 1), 14.8% of the women (35 women) had one induced abortion and 11.8% (26 women) had more than one induced abortion. Most of the women (68.4%) were wanted to stop childbearing, and 31.6% of them were planning to postpone childbearing.

**Table 1:** Descriptive features of 237 women according to the health center abortions, Diyarbakir-2004.

**Variables**	**DTFPA**
	**n (%)**

**Age (years)**	

15–19	19 (8.0)

20–24	55 (23.2)

25–29	61 (25.7)

30–39	63 (26.6)

> 40	39 (16.5)

**Education level**	

Illiterate	123 (51.9)

Literate	20 (8.4)

Primary school	60 (25.3)

Secondary school	29 (12.2)

High school or more	5 (2.1)

**Parity**	

Nulliparous	4 (1.7)

Primiparous	21 (8.9)

Para 2–4	95 (40.1)

Para 5–7	80 (33.8)

> Para 8	37 (15.6)

**Previous induced abortion**	

None	174 (73.4)

1	35 (14.8)

> 1	28 (11.8)

**Fertility choices**	

Stop childbearing	162 (68.4)

Postpone childbearing	75 (31.6)

**Marital status**	

Currently married	237 (100.0)

**Total**	237 (37.4)

### Contraceptive usage after abortion

The contraceptive usage ratios of 237 women who had induced abortion at least a year ago are shown in Table 2. Before induced abortion performed the total contraception usage (modern methods + traditional methods) rate was 44.7% and this rate increased to 80.1% at the end of one year. 180 (75.9%) women were still using one of the modern contraceptive methods. The modern method usage rate increased 62.0%. The major increasing was in IUD there was no woman with IUD before induced abortion. But now 124 (52.3%) women had IUD one year after the induced abortion performed.

**Table 2:** Distribution of currently used methods 12 months or more after induced abortion, in Diyarbakir, in 2004.

**Contraceptive Methods**	**Before**	**After**
	**n (%)**	**n (%)**

**None**	131 (55.3)	45 (19.0)

**Coitus interrupts and other traditional methods**	73 (30.8)	12 (5.1)

**Total modern methods**	33 (13.9)	180 (75.9)

*IUD*	-	124 (52.3)

*Contraceptive pills*	8 (3.4)	18 (7.6)

*Condom*	25 (10.5)	27 (11.4)

*Injectables*	-	2 (0.8)

*Tubal ligation*	-	5 (2.1)

*Vasectomy*	-	4 (1.7)

**odds ratio for total contraception (95% CI)**	0.19 (0.12–0.29)	p = 0.000

**odds ratio for modern contraception (95% CI)**	0.05 (0.13–0.25)	p = 0.000

**rate increase for total contraception usage**	%36.3	

**rate increase for modern contraception usage**	%62.0	

### Factor effecting contraceptive usage

The factors associated with contraceptive usage in women who had induced abortion one year ago were shown in Table 3. Twenty four percent of the 237 women were not using any contraceptive methods. There was no difference among age groups and education level groups regarding to contraceptive usage. According to univaried analyses nulliparous and primiparous women were more tends to not using contraceptives. Future fertility plan and previous induced abortion had no effect on contraceptive usage. If a contraceptive method was introduced immediately after abortion contraceptive usage could be higher following one year.

**Table 3:** Factors influencing contraceptive usage in women who had induced abortion, Diyarbakir-2004

	N	Women not using contraceptives (%)	p	Crude odds ratios (95% C.I.)	Adjusted odds ratio (95% C.I. for adjusted odds ratios)
**Age groups (yrs)**					

**lower than 25**	19	4 (21.1)		1	1

**25–29**	55	16 (29.1)		1,9 (0,5–6,5)	5.9 (0.6–55.1)

**30–34**	61	10 (16.4)		0,7 (0,2–3,3)	1.5 (0.4–6.4)

**35–39**	63	13 (20.6)		0,9 (0,2–4,2)	2.9 (0.8–10.7)

**more than 39**	39	14 (35.9)	0.18	2,1 (0,5–9,3)	1.9 (0.6–6.0)

**Education level**					

**Illiterate**	123	30 (24.4)		1	1

**primary school**	80	20 (25.0)		1,0 (0,5–2,1)	1.4 (0.3–5.7)

**high school**	34	7 (20.6)	0.87	0,8 (0,3–2,2)	0.8 (0.4–2.1)

**Parity**					

**nulliparous**	4	3 (75.0)		1	1

**primiparous**	21	8 (38.1)		0,2 (0,0–3,0)	0.1 (0.0–2.6)

**2–4 birth**	95	16 (16.8)		0,1 (0,0–0,8)	0.4 (0.0–3.8)

**5–7 birth**	80	19 (23.8)		0,1 (0,0–1,2)	0.9 (0.2–3.8)

**more than 7 birth**	37	11 (29.7)	0.023	0,1 (0,0–1,8)	0.5 (0.2–1.9)

**Desire for future fertility**					

**stop child bearing**	162	39 (24.1)		1	1

**postponing child bearing**	75	18 (24.0)	0.99	1,0 (0,5–1,2)	1.0 (0.3–3.1)

**A modern method was introduced immediately after abortion**					

**yes**	210	34 (16.2)		1	1

**no**	27	23 (85.2)	0.000	21,5 (6,4–79,1)	39.7 (10.8–145.2)

**Previous induced abortion**					

**none**	174	47 (27.0)		1	1

**1**	35	5 (14.3)		0,6 (0,2–1,8)	0.5 (0.2–1.9)

**>1**	28	5 (17.9)	0.197	0,6 (0,2–1,7)	2.2 (0.4–12.3)

**TOTAL**	237	57 (24.0)			

In Table 4 reasons determined by women for why they did not use contraceptives were shown. Most of the women had no answer for this manner and some of them believe that they could not get pregnant.

**Table 4:** Reasons shown by women for not using modern contraceptive methods.

**Reasons**	n	%
No reason	15	33.3

I can not get pregnant	6	13.3

I want to get pregnant	5	11.1

My husband is away from me	6	13.3

I have just give a birth	4	8.9

I am in menopause	1	2.2

I am pregnant now	4	8.9

I have divorced	4	8.9

Total	45	100.0

## Discussion

As declared at the 1994, International Conference on Population and Development in Cairo, the primary propose of the family planning programs should be to help women avoid unwanted pregnancies and achieve their fertility goals safely [11]. Every woman who decides to receive abortion reflects unwanted pregnancy and shows a failure in meeting the Cairo goals. To meet the Cairo challenge, we need better family planning counseling and higher quality family planning services. In accordance with a study conducted in Turkey, the main reasons for induced abortion are: being unable to afford raising a baby, unwillingness to raise children, willingness to postpone childbearing, advanced maternal age, risk to fetal health, and risk to maternal health [12]. In our study, we tried to answer the question why women do not use contraception; instead, they receive abortion. In addition, we tried to support the conclusion that improved post abortion family planning counseling is an important argument of reducing unwanted pregnancies.

The study conducted in a small area of Diyarbakir and the region was least developed setting so the results may not reflect the women living Diyarbakir. As this is a follow-up study 85 women couldn't be reached and this may lead selection bias and may negatively effect the results of our study.

Among our clients, 51.9% of the women were illiterate and 73.4% of them underwent abortion for the first time. The rate of women with parity over 4 was 49.4%. Age distributions, education levels, and parity of our clients were not very different from the result of Turkish National Health Survey [4]. In our study group, 55.3% of the women did not use any contraceptive method although the contraceptives are available in all primary health care centers and maternity hospitals in Turkey. In Brazil, 61% of the women who had induced abortion did not use any contraceptives and the women with higher education level, younger age, and unmarried status would tend to have abortion [13]. In USA, women undergoing abortions were likely to be young (i.e., age < 25 years), white, and unmarried; slightly more than one-half were having an abortion for the first time [14].

At the end of one year, 75,9% of the women followed by our clinic were using one of the modern contraceptive methods. Contraceptive usage was increased 36.3% for total contraceptives and 62.0% for modern methods. This difference seems to be caused by post-abortion counseling. The use of postabortion family planning significantly decreased the postabortion pregnancy rate. In Turkey similar to our study a post abortion family planning counselling programme was applied and he use of an IUD was the preferred immediate method of choice [15]. Post-abortioncounseling regarding the methods of contraception is lacking because the family planning programs in developing countries are not well designed. Findings from the study conducted in developing countries [16] indicate that post-abortion counseling for family planning should be introduced into most hospitals and that this should be seen as an integral part of the family planning program in each country. In Turkey, all contraceptive methods are available in the primary health care centers and the governmental maternal hospitals including the abortion units. In England, majority of the abortion units indicated that they provided contraceptive advice but only 74% of the units provided written information and even fewer units were able to provide a follow-up appointment within 2 weeks [17]. It was reported that the quality of counseling was critical to improving acceptance of post abortion contraception [18]. In our study, we gave the post abortion counseling by an appropriate method. All of our doctors and nurses were trained on communication skills and adult learning principles for giving more qualified counseling. Our clients were also followed by telephone call or home visits. Contraceptive methods were introduced immediately after the abortions and we monitored our clients in the meantime. These are the main factors in increased usage of contraceptives by our clients.

In public hospitals and maternity clinics, the physicians and nurses mentioned that they have never given counseling to their attendants because over crowding. Although the physicians believe in the benefit of post abortion family planning counseling, they have never had enough time to give counseling appropriately. In private clinics, gynecologists/obstetricians advised some contraceptive methods to their patients but not with proper communication skills. Additionally, in our clinic the waiting time of clients was also short. In Mexico, the doctors and nurses also agreed that the women should be well informed about the post abortion contraception, but the doctors and nurses commented not having enough time to support their clients in post abortion period and unmet contraception need was found 53% in a Mexico study [19]. This ratio is about as high as it is in Turkey. By means of better family planning counseling, unmet contraception need may also be reduced. Nevertheless 88.6% of our clients met their contraceptive need.

The contraceptive usage was reported to be affected by parity, particularly in Islamic faith [20]. Contraceptive usage was also affected with the education level [21,22]. We studied in specific group. Because of this we could not find relation between education level and contraceptive usage and we also could not find relation between parity and contraceptive usage. But the introducing of contraceptive methods immediately after induced abortion effected contraceptive usage.

This study was a pilot study and we tried to show the effect of postabortion family planning counselling to health authority of Diyarbakir city. Today most of the clinics performing abortion services was giving family planning counselling in Diyarbakir. In the study we couldn't research the effect of male partners. this is one of an other weakness of our study.

## Conclusion

In conclusion, post abortion period is the right time to introduce contraceptive advices because women are more ready to receive massages. We also concluded that introducing some advices or methods is not sufficient; more qualified counseling techniques including proper communication skills is required in post abortion period.

## Abbreviations

DTFPA: Diyarbakir Office of Turkish Family Planning Association; IUD: Intra Uterine Device; FP: Family Planning; WHO: World Health Organization; TNHS: Turkish National Health Survey.

## Competing interests

The authors declare that they have no competing interests.

## Authors' contributions

AC designed the study, carried out the area work, participated in the data collection, contribute drafting manuscript and entering the data to computer. ME designed the study, carried out the questionnaire, applied the interviews and drafted the manuscript. GS participated in the data collection and design of the study, participate to find out cases, help on statistical analyses. NA performed the abortions, conduct medical examination of women, help to drafting discussion section. All authors read and approved the final manuscript.

## Pre-publication history

The pre-publication history for this paper can be accessed here:


